# Optimal Ligand Descriptor for Pocket Recognition Based on the Beta-Shape

**DOI:** 10.1371/journal.pone.0122787

**Published:** 2015-04-02

**Authors:** Jae-Kwan Kim, Chung-In Won, Jehyun Cha, Kichun Lee, Deok-Soo Kim

**Affiliations:** 1 Voronoi Diagram Research Center, Hanyang University, Seoul, Korea; 2 School of Mechanical Engineering, Hanyang University, Seoul, Korea; 3 Department of Industrial Engineering, Hanyang University, Seoul, Korea; University of Edinburgh, UNITED KINGDOM

## Abstract

Structure-based virtual screening is one of the most important and common computational methods for the identification of predicted hit at the beginning of drug discovery. Pocket recognition and definition is frequently a prerequisite of structure-based virtual screening, reducing the search space of the predicted protein-ligand complex. In this paper, we present an optimal ligand shape descriptor for a pocket recognition algorithm based on the beta-shape, which is a derivative structure of the Voronoi diagram of atoms. We investigate six candidates for a shape descriptor for a ligand using statistical analysis: the minimum enclosing sphere, three measures from the principal component analysis of atoms, the van der Waals volume, and the beta-shape volume. Among them, the van der Waals volume of a ligand is the optimal shape descriptor for pocket recognition and best tunes the pocket recognition algorithm based on the beta-shape for efficient virtual screening. The performance of the proposed algorithm is verified by a benchmark test.

## Introduction

Drug discovery is a time consuming, costly process. One of the most critical processes in drug-discovery is identification of predicted hit where virtual screening as an *in silico* method screens a chemical library against a target protein [1–3]. For this purpose, the pharmacophore of a pocket can be used for virtual screening [4, 5]. Based on its effectiveness and the rapid accumulation of three-dimensional molecular structures, structure-based virtual screening is becoming more widespread. Over 100,000 experimentally determined biomolecular structures are cataloged in the Protein Data Bank (PDB) [6], and millions of rational biomolecular models are cataloged in the MODBASE [7], the SWISS-MODEL [8] and the PMDB [9]. Successful cases of structure-based virtual screening include Gleevec targeting a tyrosine kinase [10], Agenerase and Viracept for HIV protease [11]. Other successful cases are reviewed in [11–13].

A common approach in structure-based virtual screening is docking simulation which attempts to find the best binding of a ligand to a receptor by solving the energy minimization problem where the search space is exponential, making it hard to solve [14, 15]. In order to reduce computation, docking algorithms usually predict a potential binding site called a *pocket*, which is the concave region on the molecular boundary, to place an initial ligand for the energy minimization process [16–19].

There are three approaches in pocket recognition. The *grid-based approach* defines the lattice of the space occupied by a receptor, infers the relations among the grid points in the lattice to extract the exterior boundary of the molecule, and recognizes the depressed regions on the boundary [20–23]. A *sphere-coating approach* places a set of artificial spherical probes around the receptor and infers the relations among the probes for a pocket [24–26]. However, both approaches are rather heuristic and do not guarantee a quality solution in spite of heavy computational requirement. The *computational geometry approach* is based on the formal computational geometry theory of the proximity among atoms to recognize the receptor boundary and the shape of a pocket. The (weighted) alpha-shape based method [27, 28] and the beta-shape based method [29] belong to this category.

Most previous pocket recognition studies regarded the largest concave region on the receptor boundary as a pocket, ignoring the ligand characteristics. However, different ligands may bind to different sites on the boundary of an identical receptor. For example, c-Myc protein, which is overexpressed in the majority of human cancers, is known to have three independent binding sites corresponding to three different types of ligands: Ligands 10074-G5, 10074A4, and 10058-F4 [30] bind to 366–375, 375–385, and the 402–409 residues of c-Myc, respectively [31]. If the biggest pocket is only considered for virtual screening, drug candidates corresponding to the other two binding sites cannot be found. Hence, it is desirable to reflect the ligand characteristics during the pocket recognition process as its shape is the most important ligand characteristic. Reports for other cases are also available [32–34].

In this paper, we propose optimization of a ligand shape descriptor for pocket recognition based on the beta-shape so that the recognized pocket can be better used for virtual screening. We first present the formalization of our earlier pocket recognition algorithm [29] in the context of the beta-shape. We avoid the (weighted) alpha-shape due to the following reason. The alpha-shape was originally defined for points using the ordinary Voronoi diagram of points [35] and was used for reasoning the spatial properties of point clouds or molecular structures assuming that all atoms were of an identical size. However, poly-sized atomic model (i.e., different atom types had different radii) was more realistic for analyzing molecular structure. To reflect the size difference among different atom types, the weighted alpha-shape, which was based on the power diagram of the poly-sized atomic model, replaced the alpha-shape [36]. However, it turned out that the power diagram, and thus the weighted alpha-shape as well, was not based on the Euclidean distance but on the power distance which could be interpreted as the tangential distance from the boundary of spherical atoms. Due to this property, the topology structure of the weighted alpha-shape can be incorrect for reasoning the proximity between non-intersecting atoms and is not necessarily offset-invariant. The lack of offset-invariance causes the limitation of the weighted alpha-shape for many important applications of molecular structure.

Then, we present the optimal shape descriptor of a ligand for pocket recognition. This is based on an efficient algorithm to extract the molecular boundary using the beta-shape, a structure derived from the Voronoi diagram of the molecule [37]. Using the beta-shape and the optimized shape descriptor, effective pockets can be efficiently recognized and used for the docking algorithm called the BetaDock [38, 39]. The molecular graphics in this paper were created using BetaMol, a molecular modeling, visualization, and analysis program freely available from *http://voronoi.hanyang.ac.kr/software.htm* [40].

## Approach

### Pocket recognition using the beta-shape

**Fig 1:**
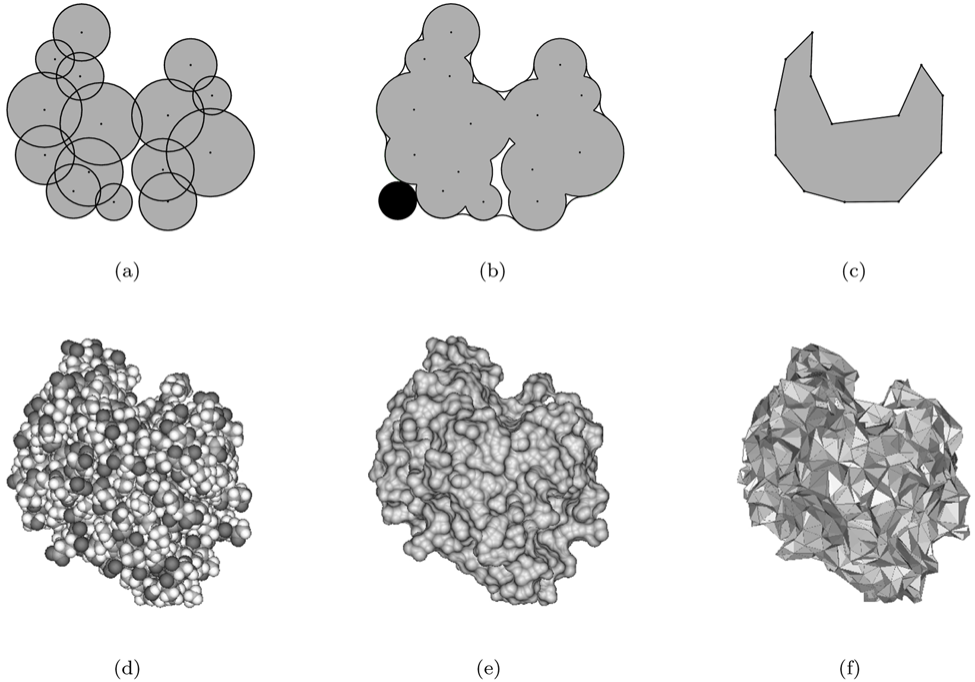
A schematic diagram of a molecule and its beta-shape. Figure drawn by using the BetaConcept[44] and BetaMol program freely available from VDRC. (a) A two-dimensional molecule, (b) A two-dimensional molecule and its Connolly surface corresponding to the red circular probe, and (c) the beta-shape corresponding to the probe, (d) the van der Waals model of a protein (PDB id 1oq5), (e) the Connolly surface for water molecule (with 1.4Å radius), and (f) the corresponding beta-shape.

For the proximity among the atoms on the molecular boundary, the concept of the beta-shape has been proposed [37]. Fig. 1(a) shows a two-dimensional molecule. Fig. 1(b) shows the Connolly surface (green curve) corresponding to the red circular probe where the radius is *β*. Suppose that the Connolly surface is straightened by substituting the straight edges for the circular arcs and the planar triangles for the spherical triangles where their vertices are the centers of the related atoms. The straightened object bounded by the planar facets is the *beta-shape* of the molecule. Fig. 1(c) shows the beta-shape of a molecule corresponding to the red circular probe in Fig. 1(b). The beta-shape concisely provides the precise proximity among the atoms on the molecular boundary with respect to the probe. Fig. 1(d), (e), and (f) show the van der Waals model of a protein (PDB id 1oq5), its Connolly surface for water molecule with 1.4Å radius, and the corresponding beta-shape. We note here that the beta-shape is efficiently computed from the *quasi triangulation* which is the dual structure of the *Voronoi diagram of atoms*. The details are reported in [37, 41–43] and readers are recommended to download the BetaConcept program from VDRC (http://voronoi.hanyang.ac.kr) to explore the properties of the beta-shape.

**Fig 2:**
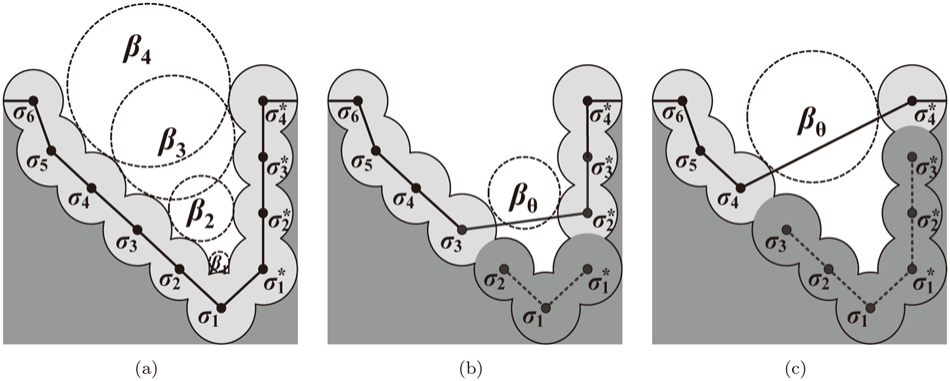
The idea of pocket recognition using the beta-shape. (a) Empty tangent balls defining the exposure intervals of each atom on the boundary. (b) The pocket {*σ*_1_, *σ*_2_, $\sigma_{1}^{*}$} where *β*_2_ < *β*_*θ*_ ≤ *β*_3_. (c) The pocket {*σ*_1_, *σ*_2_, *σ*_3_, $\sigma_{1}^{*}$, $\sigma_{2}^{*}$, $\sigma_{3}^{*}$} where *β*_3_ < *β*_*θ*_ ≤ *β*_4_.

Fig. 2 shows a two-dimensional schematic diagram showing the idea of pocket recognition using the beta-shape. Suppose that the figure depicts a subset of the beta-shape corresponding to the probe of water. Consider that the small circle *σ* or σ* is an atom on the molecular boundary and the shaded region is the molecular interior. The atoms on the slanted wall in the left are numbered *σ*_1_ through *σ*_6_, and those on the vertical wall are numbered $\sigma_{1}^{*}$ through $\sigma_{4}^{*}$. There are four dotted circles *β*_1_, *β*_2_, *β*_3_ and *β*_4_ in Fig. 2(a) where each is in contact with the boundary of the three atoms. For convenience, suppose that *β*_1_, *β*_2_, *β*_3_ and *β*_4_ also denote the radii of the corresponding circles where 0 ≤ *β*_1_ < *β*_2_ < *β*_3_ < *β*_4_. Let *π* be a spherical open probe with the radius *β*_*π*_.

In Fig. 2(a), the smallest circle *β*_1_ is in contact with *σ*_1_, *σ*_2_ and $\sigma_{1}^{*}$. Consider a probe *π* smaller than *β*_1_ (i.e., *β*_*π*_ ≤ *β*_1_). Then, *π* can touch the boundary of all atoms implying that all atoms are exposed to *π*. However, if *β*_*π*_ is greater than *β*_1_, *π* can no longer touch *σ*_1_ and *σ*_1_ is not exposed to *π*. Hence, *σ*_1_ is exposed when 0 ≤ *β*_*π*_ ≤ *β*_1_, and the interval [0,*β*_1_] is called the *exposure interval* for *σ*_1_. Consider *β*_2_, which is in contact with the three atoms *σ*_2_, *σ*_3_ and $\sigma_{2}^{*}$. Then, *σ*_2_ is similarly exposed when 0 ≤ *β* ≤ *β*_2_. The exposure interval of *σ*_3_ is [0,*β*_3_]. A similar observation holds for the other atoms. Therefore, each boundary atom is associated with an exposure interval.

Fig. 2(b) and (c) illustrate how to use the exposure interval in pocket recognition. Let *β*_*θ*_ be the threshold value to recognize a pocket. Suppose that *β*_2_ < *β*_*θ*_ ≤ *β*_3_. This implies that the atoms *σ*_1_ and *σ*_2_ ($\sigma_{1}^{*}$ and $\sigma_{2}^{*}$ as well) are not exposed to *π* when *β*_*π*_ = *β*_*θ*_. Then, the boundary of the beta-shape corresponding to *π* = *β*_*θ*_ is shown as the solid polyline in Fig. 2(b). Hence, the boundary no longer includes the three atoms *σ*_1_, *σ*_2_ and $\sigma_{1}^{*}$ and the depressed, buried region consisting of *σ*_1_, *σ*_2_ and $\sigma_{1}^{*}$ can be regarded as a pocket. Therefore, the atoms that constitute a pocket can be easily identified by checking the exposure interval of each atom. Fig. 2(c) shows a larger pocket. A lager *β*_*θ*_ tends to define a larger pocket and a smaller *β*_*θ*_ tends to define a smaller pocket. As different *β*_*θ*_ values define different pockets, it is important to find the optimal value of *β*_*θ*_. The threshold *β*_*θ*_ is essential for the shape and size of the pockets. For details, see [45].

### L-descriptor: descriptor of the ligand shape

Drug-like ligands ordinarily consist of 20 to 70 atoms [46] where each can have various conformations [47]. The conformation of a ligand instance affects the binding between the ligand and its receptor, and the primary factor of the binding is the ligand shape. Therefore, an appropriate consideration of the ligand shape is necessary. There are algorithms for computing the possible ligand conformations so that each conformation can be treated as a ligand instance in virtual screening [48]. The pocket recognition algorithm above uses the threshold *β*_*θ*_ whose optimal value for a given pair of ligands and receptors should be inferred to form the measure of the ligand shape. We call this measure the *L-descriptor*.

**Fig 3:**
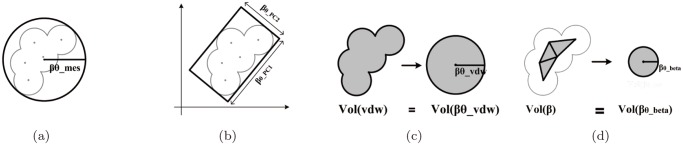
L-descriptor types in the plane. (a) The minimum enclosing sphere and *β*_*θ*__mes, (b) the bounding box by PCA, *β*_*θ*__PC1, and *β*_*θ*__PC2, (c) the van der Waals model of the ligand and *β*_*θ*__vdW, and (d) the beta-shape of the ligand and *β*_*θ*__beta.

**Fig 4:**
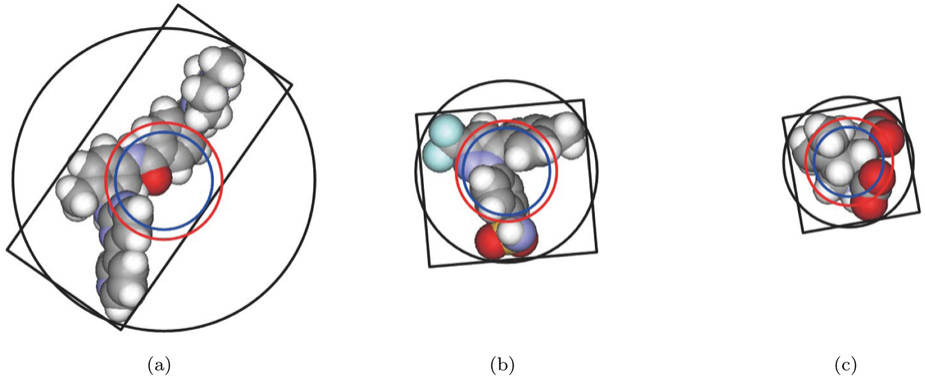
Some of the proposed L-descriptor types. The black circle denotes the minimum enclosing sphere; the red circle denotes the sphere whose volume is identical to the volume of the van der Waals model of the ligand; the blue circle denotes the sphere whose volume is identical to the volume of the beta-shape; the black rectangle denotes the bounding box of the PCA analysis. The PDB accession codes that contains the complex with the shown ligands are as follows: (a)1t46, (b)1oq5, and (c) 1tt1.

We examine six types of L-descriptor for a ligand: *β*_*θ*__*mes*, *β*_*θ*__*PC*1, *β*_*θ*__*PC*2, *β*_*θ*__*PC*3, *β*_*θ*__*vdW* and *β*_*θ*__*beta*. The *β*_*θ*__*mes* is the radius of the minimum enclosing sphere (mes), which is the smallest sphere that contains all the ligand atoms (Fig. 3(a)). The values of *β*_*θ*__*PC*1, *β*_*θ*__*PC*2 and *β*_*θ*__*PC*3 are obtained from the bounding box of a ligand that is computed by the principal component analysis (PCA) [49]. Let PC1 be the first principal component denoting the greatest variance of the data set. Similarly, let PC2 and PC3 be the second and the third principal components denoting the second and third greatest variance, respectively. Then, the length of each edge of the PCA-induced bounding-box is used as *β*_*θ*__*PC*1, *β*_*θ*__*PC*2, or *β*_*θ*__*PC*3. See Fig. 3(b) for examples of *β*_*θ*__*PC*1 and *β*_*θ*__*PC*2 in the plane. Two volume measures are also investigated. Let *Vol*(*vdW*) be the volume of the vdW-model of a ligand. Consider a sphere whose volume is also *Vol*(*vdW*). Then, the radius of the sphere is *β*_*θ*__*vdW* (Fig. 3(c)). For computation of *Vol*(*vdW*), refer to [50]. Let *Vol*(*β*) be the volume of the beta-shape corresponding to the spherical probe of a water molecule. Then, the radius of the sphere with the volume *Vol*(*β*) is *β*_*θ*__*beta* (Fig. 3(d)). Fig. 4 shows the three-dimensional counterpart of the L-descriptors for three ligands found from protein complexes in PDB.

## Methods

### Definition of an optimal pocket

**Fig 5:**
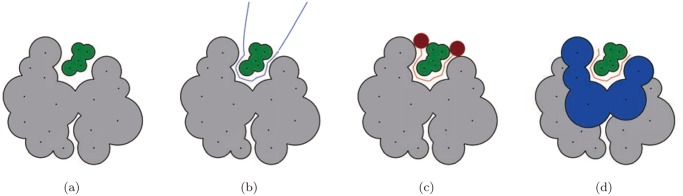
The interaction interface (IIF) of a two-dimensional molecule complex and the optimal pocket defined by *IIF*. **The gray and green objects are a receptor molecule *M*^*R*^ and a ligand molecule *M*^*L*^, respectively.** (a) A two-dimensional molecule complex, (b) *IIF* ^∞^ shown as the blue curve, (c) *IIF* shown as the red curve trimmed by the red circle, and (d) the optimal pocket consisting of the five blue atoms and *IIF*.

Consider a complex consisting of a receptor molecule *M* ^*R*^ (the gray object in Fig. 5(a)) and its bound ligand molecule *M* ^*L*^ (the green object the same figure) where both are defined by atom sets. Let ∂*M* ^*R*^ be the boundary of the van der Waals model of *M* ^*R*^ and *d*(*q*,*M* ^*R*^) the minimum Euclidean distance between two points *q* and *x* ∈ ∂*M* ^*R*^. ∂*M* ^*L*^ and *dist*(*q*,*M* ^*L*^) are similarly defined. Let *IIF* ^∞^ = {*q*_1_,*q*_2_,*q*_3_,…} be the surface (the blue curve in Fig. 5(b)) which is the locus of *q*_*i*_ where *dist*(*q*_*i*_,*M* ^*R*^) = *dist*(*q*_*i*_,*M* ^*L*^). In other words, *IIF* ^∞^ is the mid-surface between *M* ^*R*^ and *M* ^*L*^ emanating to infinity. Let *IIF* ⊂ *IIF* ^∞^ be the trimmed surface (the red curve in Fig. 5(d)) of *IIF* ^∞^ using the probe of a water molecule as a cutter (the red ball in Fig. 5(c)) [51]. Then, *IIF* is called the *interaction interface* between *M* ^*R*^ and *M* ^*L*^. Let Π ⊂ *M* ^*R*^ be the set of receptor atoms (the blue five atoms in Fig. 5(d)) which defines *IIF*. Then, we call Π the *optimal pocket* in this paper. Π is called optimal in the sense that a complex consisting of a receptor and a ligand is crystalized, and its structure is solved in its entirety. For the details, see [52].

### Evaluation of a recognized pocket

In a binary decision problem, a decision made by a classifier can be represented in a confusion matrix [53]. Recall that Π denotes the optimal pocket. Let Π^*c*^ = *B*−Π where *B* is the set of atoms on the receptor boundary. In other words, Π^*c*^ is the boundary atoms except those in the optimal pocket. Let $\hat{\Pi }$ be the recognized pocket by the proposed algorithm. Then, ${\hat{\Pi }}^{c}=B-\hat{\Pi }$ is the boundary atoms except those in the recognized pocket.

We can now define the confusion matrix for pocket recognition as in Table 1. The atoms in $\Pi \cap \hat{\Pi }$ are called true positive (*T* ^+^); The atoms in $\Pi^{c}\cap {\hat{\Pi }}^{c}$ are called true negative (*T* ^−^); The atoms in $\Pi^{c}\cap \hat{\Pi }$ are called false positive (*F* ^+^); The atoms in $\Pi \cap {\hat{\Pi }}^{c}$ are called false negative (*F* ^−^). Hence, *true positive*(*T* ^+^) refers to the positive atoms correctly recognized as positive; *False positive*(*F* ^+^) refers to the negative atoms incorrectly recognized as positive; *True negative*(*T* ^−^) refers to the negative atoms correctly recognized as negative; *False negative*(*F* ^−^) refers to the positive atoms incorrectly recognized as negative.

**Table 1:** Confusion matrix for pocket evaluation.

	**In recognized pocket ($\hat{\Pi }$)**	**Not in recognized pocket (${\hat{\Pi }}^{c}$)**
**In optimal pocket (Π)**	True Positive (*T* ^+^)	False Negative (*F* ^−^)
**Not in optimal pocket (Π^*c*^)**	False Positive (*F* ^+^)	True Negative (*T* ^−^)

Given the confusion matrix, various metrics can be defined for the evaluation of the quality of a recognized pocket. The true positive rate, *TPR*, is the proportion of the correct atoms in the recognized pocket (*T* ^+^) against the atoms in the optimal pocket (both *T* ^+^ and *F* ^−^). *TPR* is also referred to as the *recall rate* *R*, or the *sensitivity* *S*. The false positive rate, *FPR*, is the proportion of the incorrect atoms of the recognized pocket (*F* ^+^) against the atoms which do not belong to the optimal pocket (both *T* ^−^ and *F* ^+^). The *specificity*, *SP*, is the proportion of the correct atoms not in the recognized pocket (*T* ^−^) against the atoms not in the optimal pocket (both *T* ^−^ and *F* ^+^). The *precision*, *P*, is the proportion of the correct atoms in the recognized pocket (*T* ^+^) against the atoms in the recognized pocket (both *T* ^+^ and *F* ^+^). The *accuracy*, *AC*, is the proportion of correct atoms in the recognized pocket (both *T* ^+^ and *T* ^−^) against all atoms in the boundary *B*. In this paper, these are called the *primary metrics* from the confusion matrix and summarized in Table 2.

**Table 2:** Primary metrics of the confusion matrix.

**Primary metric**	**Equation**
True Positive Rate (TPR)	$TPR=\frac{T^{+}}{T^{+}+F^{-}}=\frac{n(\Pi \cap \hat{\Pi })}{n(\Pi )}$
False Positive Rate (FPR)	$FPR=\frac{F^{+}}{T^{-}+F^{+}}=\frac{n(\Pi^{c}\cap \hat{\Pi })}{n(\Pi^{c})}$
Precision (P)	$P=\frac{T^{+}}{T^{+}+F^{+}}=\frac{n(\Pi \cap \hat{\Pi })}{n(\hat{\Pi })}$
Specificity (SP)	$SP=\frac{T^{-}}{T^{-}+F^{+}}=\frac{n(\Pi^{c}\cap {\hat{\Pi }}^{c})}{n(\Pi^{c})}=1-FPR$
Accuracy (AC)	$AC=\frac{T^{+}+T^{-}}{T^{+}+F^{+}+T^{-}+F^{-}}=\frac{n((\Pi \cap \hat{\Pi })\cup (\Pi^{c}\cap {\hat{\Pi }}^{c}))}{n(B)}$
Sensitivity (S) = Recall (R)	*S* = *R* = *TPR*

There are trade-offs among the primary metrics. A good recognized pocket should have high *TPR* and low *FPR* values. An overestimated, large pocket tends to have higher values for both *TPR* and *FPR* because there can be both many correctly identified atoms and many incorrectly identified atoms at the same time. An underestimated, small pocket tends to have a low *FPR* value (because the pocket size is small and thus there is a lower chance to have incorrect atoms) and a low *TPR* value (because the chance to have correct atoms is also lower). This trade-off is conveniently represented in the Receiver Operator Characteristic (ROC) graph which is useful for visualizing the performance of classifiers [54]. In the ROC-graph, the horizontal and vertical axes denote *FPR* and *TPR*, respectively. Hence, the coordinate (*FPR* = 0, *TPR* = 1) denotes the perfect pocket recognition. In the ROC-graph, the more upper-left a coordinate is, the better the performance. Given the operating points in the ROC-graph, a smooth ROC-curve can be computed with the assumption of binormal distribution. Then, the *area under the ROC-curve*, *AUC*, is a measure combining both *TPR* and *FPR* that is interpreted as the average sensitivity over all of the specificity range. In other words, *AUC* is the probability that a pocket recognizer will select a randomly chosen pocket atom higher than a randomly chosen atom not in a pocket.

It is usual that the number of atoms that do not belong to the optimal pocket significantly exceeds the number of atoms belonging to the optimal pocket. In other words, *n*(Π^*c*^) >> *n*(Π). Since $\Pi^{c}\cap \hat{\Pi }\subseteq \hat{\Pi }$ and $\hat{\Pi }\approx \Pi$, the numerator of FPR is usually significantly smaller than its denominator. Thus, even a large change in *F* ^+^ does not result in a significant change in the *FPR*. Hence, in pocket recognition, a ROC-graph tends be optimistic in that most recognized pockets and algorithms are likely to have low *FPR* regardless of the performance in reality.

The PR-graph denotes the coordinate system where the horizontal and vertical axes are the recall *R* and the precision *P*, respectively. Note that the precision *P* captures the size of the correctly recognized pocket because $\Pi \cap \hat{\Pi }\subseteq \Pi$ and $\Pi \approx \hat{\Pi }$. In the PR-graph, there is a trade-off between *R* and *P*. If all the atoms of an optimal pocket are perfectly predicted, *R* = 1, and if no atom of an optimal pocket is predicted at all, *R* = 0. If all the atoms of a recognized pocket are correct (i.e., there is no noise atoms in a recognized pocket), *P* = 1, and if all the atoms of a recognized pocket are noise atoms, *P* = 0. Hence, perfect pocket recognition occurs at the coordinates (*R* = 1, *P* = 1). Therefore, the more upper-right a coordinate is, the better the performance.

An overestimated, large pocket tends to have a high *R* (due to having many correct atoms) but a small *P* (because there are many noise atoms as well). On the other hand, an underestimated, small pocket tends to have a high *P* (because the size is small and it has lower chance to have noise atoms) but has a low *R* (because the chance to have correct atoms is lower).

Normalized Mutual Information [55], *NMI*, is a measure of information transmission which is based on Shannon’s Entropy. Entropy measures are widely used in comparing true data with predicted data. Among those possible measures, entropy measures focus on the amount of the cross-section together with the match of total amount. Given a confusion matrix, the following four entropy values can be defined: the row entropy *H*(*x*), the column entropy *H*(*y*), and two conditional entropies *H*(*x*∣*y*) and *H*(*y*∣*x*)

$$\begin{array}{c} H\left(x\right)=-\sum_{i}p_{i}\log_{2}p_{i}, \end{array}$$ (1)

$$\begin{array}{c} H\left(y\right)=-\sum_{j}p_{j}\log_{2}p_{j}, \end{array}$$ (2)

$$\begin{array}{c} H\left(x|y\right)=\sum_{j}p_{j}\left[-\sum_{i}\frac{p_{ij}}{p_{i}}\log_{2}\frac{p_{ij}}{p_{j}}\right], \end{array}$$ (3)

$$\begin{array}{c} H\left(y|x\right)=\sum_{i}p_{i}\left[-\sum_{j}\frac{p_{ij}}{p_{j}}\log_{2}\frac{p_{ij}}{p_{i}}\right] \end{array}$$ (4)

where *p*_*i*_ and *p*_*j*_ represent the empirical probabilities of the predicted and true examples, respectively, and *p*_*ij*_ is their joint probability. Then, *NMI* is defined as

$$\begin{array}{c} NMI=\frac{H(x)-H(x|y)}{H(x)}. \end{array}$$ (5)

The *NMI* contains more details of the confusion matrix which is not accounted for by other metrics [56]. The likelihood ratio test, *LR*, is a related metric that statistically compares the maximum likelihood of an unrestricted model with a restricted model [57] and is defined as

$$\begin{array}{c} LR=2\sum_{i,j}Observed\log\left[\frac{Observed}{Expected}\right] \end{array}$$ (6)

implying

$$\begin{array}{ccc} LR & = & 2\{N\log N+T^{+}\log T^{+}+F^{-}\log F^{-}+T^{-}\log T^{-}+F^{+}\log F^{+}\\ & & -\;\left(T^{+}+F^{+}\right)\log\left(T^{+}+F^{+}\right)-\left(T^{+}+F^{-}\right)\log\left(T^{+}+F^{-}\right)\\ & & -\;\left(T^{-}+F^{+}\right)\log\left(T^{-}+F^{+}\right)-\left(T^{-}+F^{-}\right)\log\left(T^{-}+F^{-}\right)\}. \end{array}$$ (7)

Both the *LR* and *NMI* are based on information entropy, which is loosely similar to the variance of the entries in the confusion table Table 2. Note also that the metric derived from the information entropy is independent of the ligand size.

In addition, we tested eleven more secondary metrics for the proposed six L-descriptors in Table 3: four based on ROC, four based on the precision, and three based on the ordinal association. The four metrics related to ROC graph are as follows: The balanced accuracy (*BA*) is defined as the numerical mean of S and *SP*[58]. The geometric mean 2 (*G*2) is the geometric mean of *S* and *SP*[59]. The Euclidean distance from an ideal classification (*ED*) is the combination of *S* and *SP* that measures the distance from an ideal classification in ROC space, where *S* and *SP* both equal one [56]. Youden index (*YI*) is the sum of the *S* and *SP* minus one and is a measure of goodness for diagnostic tests [60].

**Table 3:** Evaluation metrics.

	**Secondary metric**	**Equation**
***ROC-based metrics***	Balanced accuracy (BA)	$BA=\frac{S+SP}{2}$
	Geometric mean 2 (G2)	$G2=\sqrt{S\times SP}$
	Euclidean distance (ED)	$ED=\sqrt{{\left(S-1\right)}^{2}+{\left(SP-1\right)}^{2}}$
	Youden index (YI)	*YI* = *S*+*SP*−1
***Precision-based metrics***	F-measure (*f*)	$f=\frac{2\times S\times P}{S+P}$
	Geometric mean 1 (G1)	$G1=\sqrt{S\times P}$
	Predictive summary index (PSI)	*PSI* = *NPV*+*P*−1
	Negative Predictive Value (NPV)	$NPV=\frac{T^{-}}{T^{-}+F^{-}}=\frac{n(Op^{c}\cap Rp^{c})}{n(Rp^{c})}$
***Ordinal association metrics***	Gamma (*γ*)	$\gamma =\frac{T^{+}\cdot T^{-}-F^{+}\cdot F^{-}}{T^{+}\cdot T^{-}+F^{+}\cdot F^{-}}$
	Tau-b (*τ*_*b*_)	$\tau_{b}=\frac{T^{+}\cdot T^{-}-F^{+}\cdot F^{-}}{\sqrt{\left(T^{+}+F^{-})(T^{-}+F^{+})(T^{+}+F^{+})(T^{-}+F^{-}\right)}}$
	Tau-c (*τ*_*c*_)	$\tau_{c}=\frac{4\left(T^{+}\cdot T^{-}-F^{+}\cdot F^{-}\right)}{{\left(T^{+}+T^{-}+F^{+}+F^{-}\right)}^{2}}$

The four metrics related to PR graph are as follows: The F-measure (*f*) is a harmonic mean of *P* and *S* and was first used by Lewis and Gale for assessing text classification effectiveness and [61]. The geometric mean 1 (*G*1) is the geometric mean of *P* and *S*[59]. The predictive summary index (*PSI*) is the sum of *P* and *NPV* minus one and was developed as a measure of goodness for diagnostic tests [62]. The negative predictive value (*NPV*) is the proportion of the correct atoms out of the computed pockets (*T* ^−^) against the atoms out of the computed pocket (both *T* ^−^ and *F* ^−^).

The ordinal association metrics have been used for the analysis of cross classifications with ordinal categories. The gamma (*γ*) is the estimated difference between the probability of concordance and the probability of discordance and has a range 1 ≤ *γ* ≤ 1 [63]. The Kendall’s *τ*_*b*_ makes an adjustment for ties when it measures the proportion of concordant and discordant pairs. The Kendall’s *τ*_*c*_ is a variant of *τ*_*b*_, which makes an adjustment for table size in addition to a correction for ties [64]. Both *τ*_*b*_ and *τ*_*c*_ has range 1 ≤ *τ*_*b*_,*τ*_*c*_ ≤ 1.

From the results of the ROC-graph and PR-graph, it is important to note the following: i) The *AUC* of ROC-curve can mislead because the curve cannot reflect the low sensitivity of smaller L-descriptor, and ii) the *AUC* of PR-curve can also mislead because the curve cannot reflect the low precision of larger L-descriptor. This phenomenon resides in the various secondary metrics based on the ROC-graph and PR-graph.

**Fig 6:**
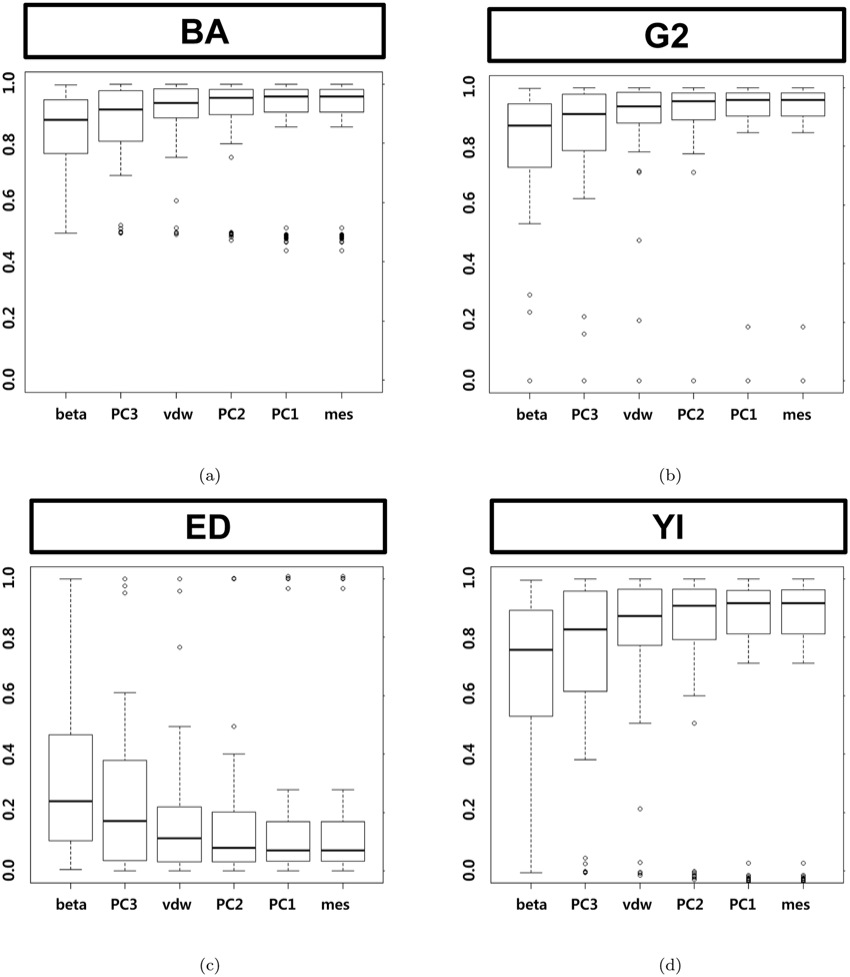
Box plots by ROC-based metrics of the six shape descriptors. (a) Balanced accuracy, (b) geometric mean 2, (c) Euclidean distance and (d) Youden index.

**Fig 7:**
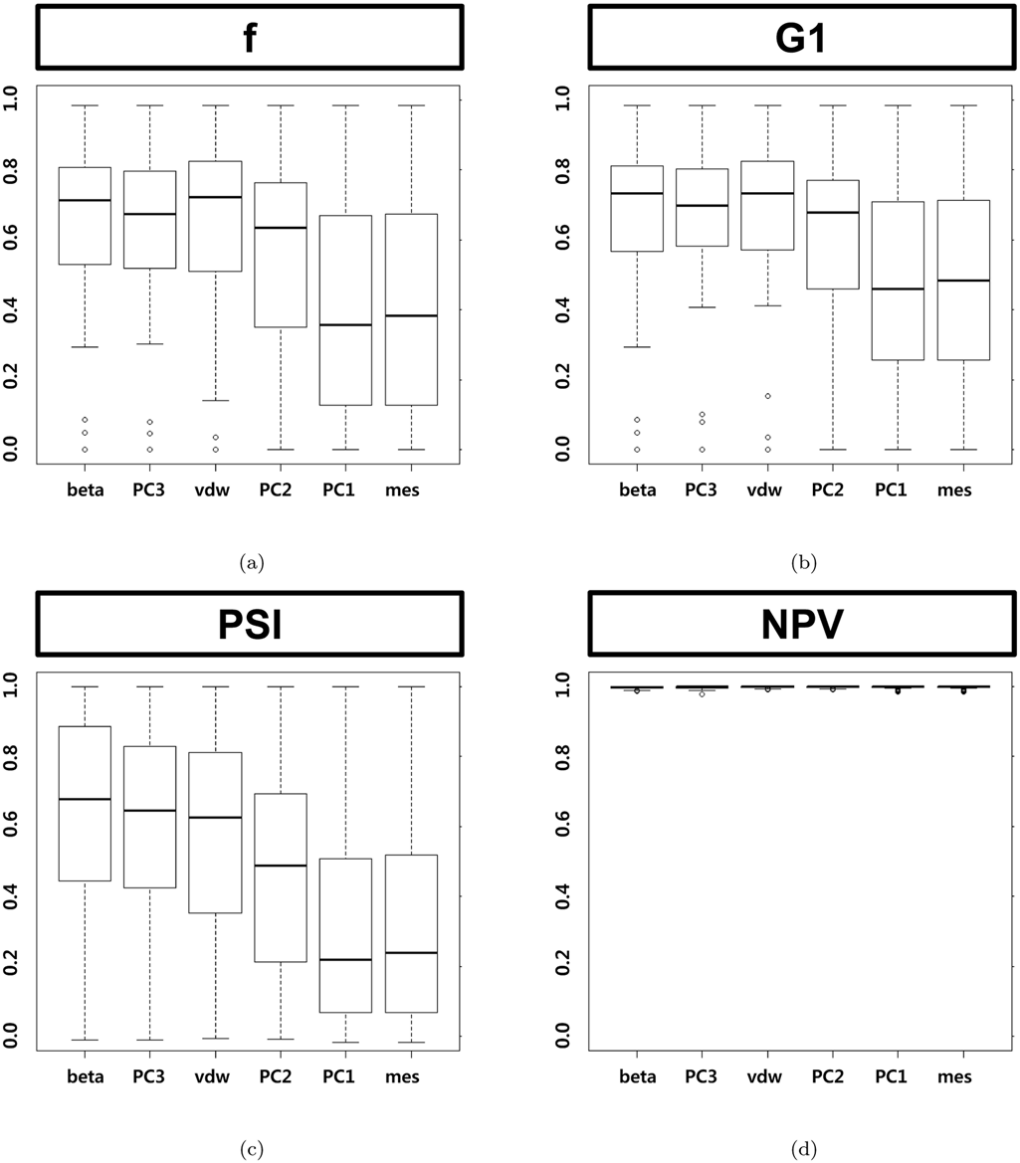
Box plots by Precision-based metrics of the six shape descriptors. (a) F-measure, (b) geometric mean 1 and (c) predictive summary index (d) negative predictive value.

Fig. 6 shows the results of the ROC-based metrics which is based on sensitivity and specificity. Fig. 7 shows the results of metrics based on precision. These PR-based metrics mislead because the metrics cannot reflect the low precision of larger L-descriptor. Negative predictive value cannot discriminate among the L-descriptor types at all, because an optimal pocket has larger negative cases than positive cases. In all metrics, it turns out that the van der Waals volume consistently belongs to the group of L-descriptors showing better performance.

## Results

### Experimental materials and methods

The experiment was done using the Astex Diverse Set (ADS) consisting of 85 high resolution protein-ligand complexes containing drug-like compounds [65]. The optimal pocket Π of each receptor was computed from the bound complex, and the corresponding recognized pocket $\hat{\Pi }$ was computed from each receptor after the bound ligand was removed.

Consider an effective, optimal pocket related to a given ligand, and suppose that there is more than one depressed region on the receptor boundary that can be considered as a *pocket candidate*. Obviously, the larger the number of pockets used in the docking simulation, the better the solution quality, and the more time a computation takes. In this experiment, we assumed that the optimal pocket corresponds to one of the five biggest pocket candidates in terms of the number of atoms belonging to each pocket candidate. In fact, in most of the cases in our experiment, the optimal pocket belonged to one of the two biggest pocket candidates.

**Fig 8:**
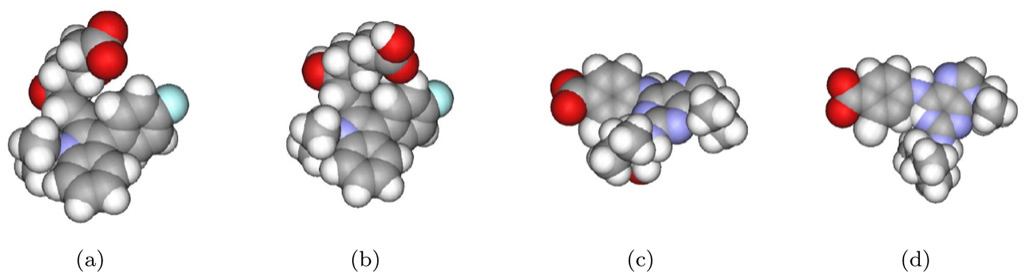
Two different conformations of two ligands: the native state and the minimum energy state. **The minimized energy conformation is calculated by MM2 in ChemOffice software.** (a) and (b) the native and the minimum energy conformations of 1hwi, respectively; (c) and (d) those of 1v0p.

A ligand may have rotational bonds that can generate various conformations. In this experiment, we used two conformations for each ligand to check the effect of a ligand’s conformation change: i) the native conformation found in the crystal structure and ii) the minimum energy conformation that was calculated by the MM2 method using ChemOffice software [66]. Fig. 8 shows two such examples.

### L-descriptors and ligand size

**Fig 9:**
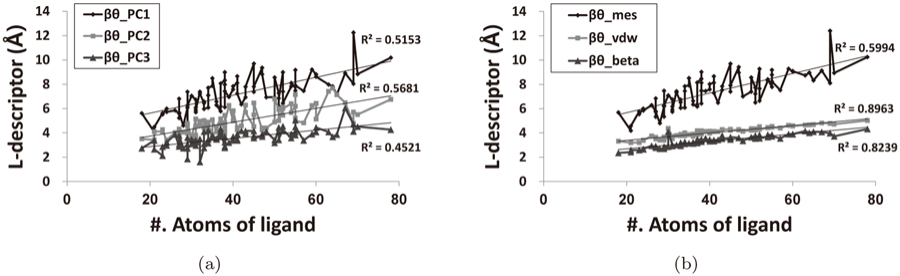
L-descriptor curves with respect to the ligand size. R^2^ (the coefficient of determination) is a statistical measure of how close the data are to the fitted regression line. The p-values of the six linear regressions are all less than 10^−11^.

Fig. 9 shows the curves for the L-descriptors vs. the ligands ordered in their sizes. The six L-descriptors are divided into two graphs: Fig. 9(a) for the PC1, PC2, and PC3; Fig. 9(b) for the minimum enclosing sphere, the van der Waals volume, and the beta-shape volume. The L-descriptors tend to increase with respect to the ligand size, and their average values are in the following order (Within the parentheses are the averages):

$$\begin{array}{ccc} & & \beta_{\theta }\_beta\left(3.35\right)<\beta_{\theta }\_PC3\left(3.60\right)<\beta_{\theta }\_vdW\left(4.04\right)\\ & & \;<\beta_{\theta }\_PC2\left(4.96\right)<\beta_{\theta }\_PC1\left(7.21\right)<\beta_{\theta }\_mes\left(7.41\right). \end{array}$$ (8)

When *β*_*X*_ < *β*_*Y*_ in Equation (8), we say that *β*_*X*_ is *smaller* than *β*_*Y*_ and *β*_*Y*_ is *bigger* than *β*_*X*_.

### Pocket evaluation

**Fig 10:**
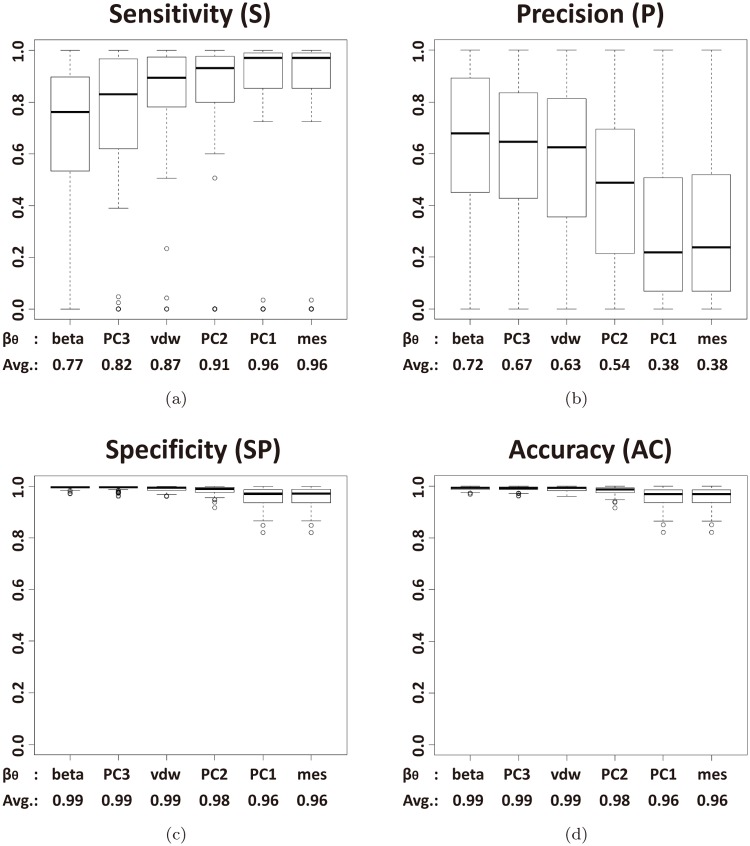
Box plots by primary metrics of the six types of L-descriptor. (a) Sensitivity, (b) precision, (c) specificity, and (d) accuracy.

Fig. 10 compares the six L-descriptor types with four primary metrics; the sensitivity *S*, the precision *P*, the specificity *SP*, and the accuracy *AC*. The horizontal axis denotes the L-descriptors in the order given in Equation (8). The vertical axis denotes the metric values. Fig. 10(a) shows that a bigger L-descriptor tends to produce a higher sensitivity value than a smaller one. This implies that a bigger L-descriptor tends to produce a larger recognized pocket which has a higher chance to have more correct atoms. On the other hand, Fig. 10(b) shows that a smaller L-descriptor tends to have a higher value of precision than a bigger one. This implies that a larger pocket has a higher chance to have incorrect atoms in a recognized pocket. This observation thus shows the trade-offs among the sensitivity and the precision. Fig. 10(c) and (d) shows that the specificity and the accuracy cannot properly discriminate the L-descriptor types.

**Fig 11:**
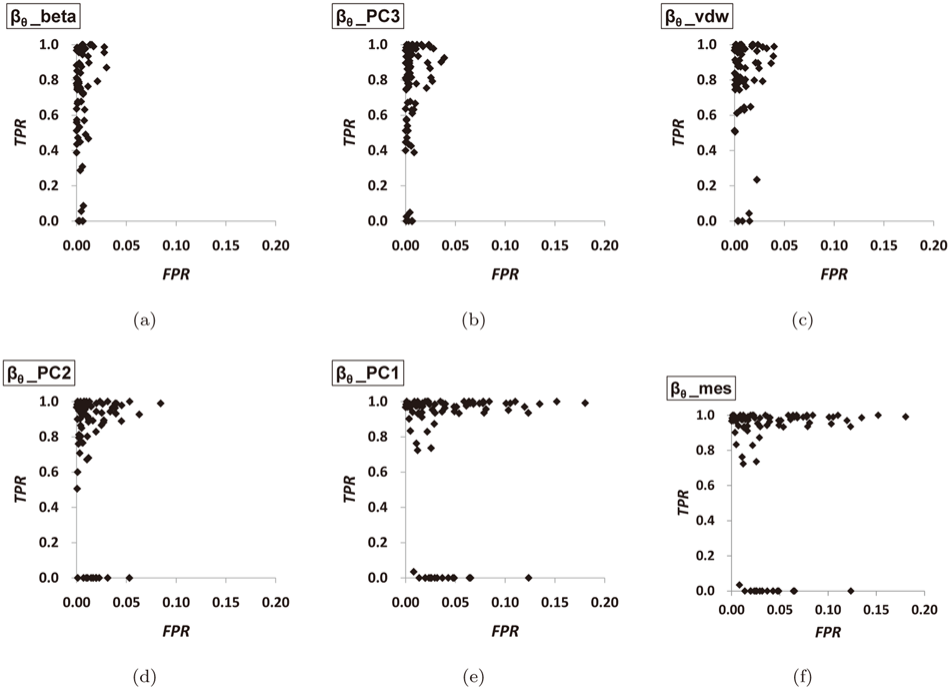
The ROC-graph of the L-descriptors. (a) the beta-shape volume, (b) the PC3, (c) the van der Waals volume, (d) the PC2, (e) the PC1, and (f) the minimum enclosing sphere.

**Fig 12:**
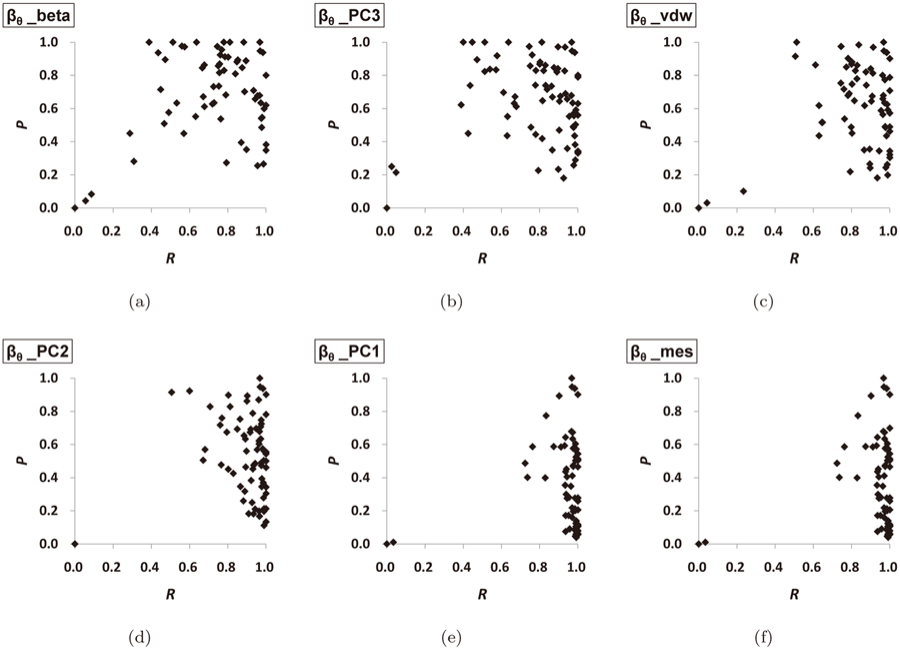
The PR-graph of the L-descriptors. (a) the beta-shape volume, (b) the PC3, (c) the van der Waals volume, (d) the PC2, (e) the PC1, and (f) the minimum enclosing sphere.

Fig. 11 and Fig. 12 show the ROC-graphs and the PR-graphs of the six L-descriptor types, respectively, in the order as before. In the ROC-graphs in Fig. 11, the *FPR* tends to be small because there are many boundary atoms which do not belong to the optimal pocket. Note that the window of the horizontal-axis is given between 0 and 0.2. From these graphs, we observe that Fig. 11(c) and (d) shows the best distribution of the *FPR* and *TPR* values. Fig. 11(a) and (b) shows rather widely distributed *TPR* values and Fig. 11(e) and (f) shows rather widely distributed *FPR* values. Recall that the perfect match occurs at the point (*FPR* = 0,*TPR* = 1). In the PR-graphs in Fig. 12, we observe that Fig. 12(c) (*β*_*θ*__*vdW*) and (d) (*β*_*θ*__*PC*2) show the best distribution of the *R* and *P* values. Fig. 12(a) and (b) shows rather widely distributed *R* values and Fig. 12(e) and (f) shows that the *P* values are rather downward distributed. Recall that a perfect match occurs at the point (*R* = 1,*P* = 1).

**Fig 13:**
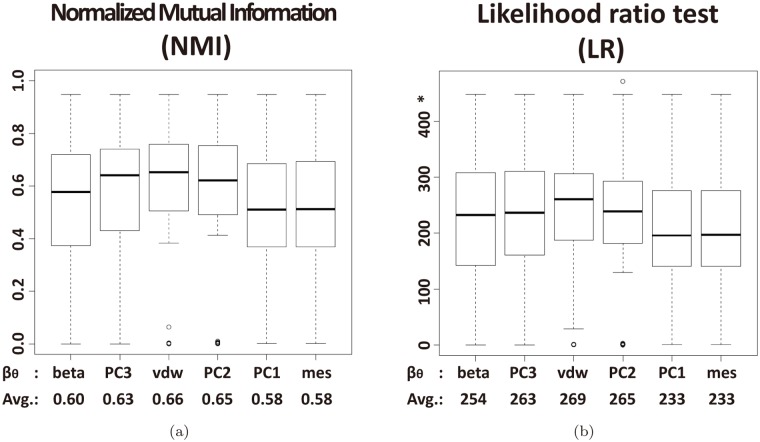
Box plots by entropy-based metrics of the six types of L-descriptor. (a) normalized mutual information and (b) Likelihood ratio. *Note that the y-axis scale of the *LR* plot is different from the *NMI* plot’s.

Fig. 13(a) and (b) shows the normalized mutual information *NMI* and the likelihood ratio *LR*, respectively, and both suggest that *β*_*θ*__*vdw* and *β*_*θ*__*PC*2 are better than the others. The value of *β*_*θ*__*vdW* is again slightly better than *β*_*θ*__*PC*2. From a statistical view point, however, it is difficult to make a clear statement of their superiority. In this regard, we performed further statistical tests with additional eleven metrics and summarized the result in S1 Table of the supplementary material. The test clearly shows that the van der Waals volume of L-descriptors is consistently better measure than the others. For details, see the “Section 4. Secondary metrics tested” in the Supplementary material.

### Optimal L-descriptor: the van der Waals volume

**Fig 14:**
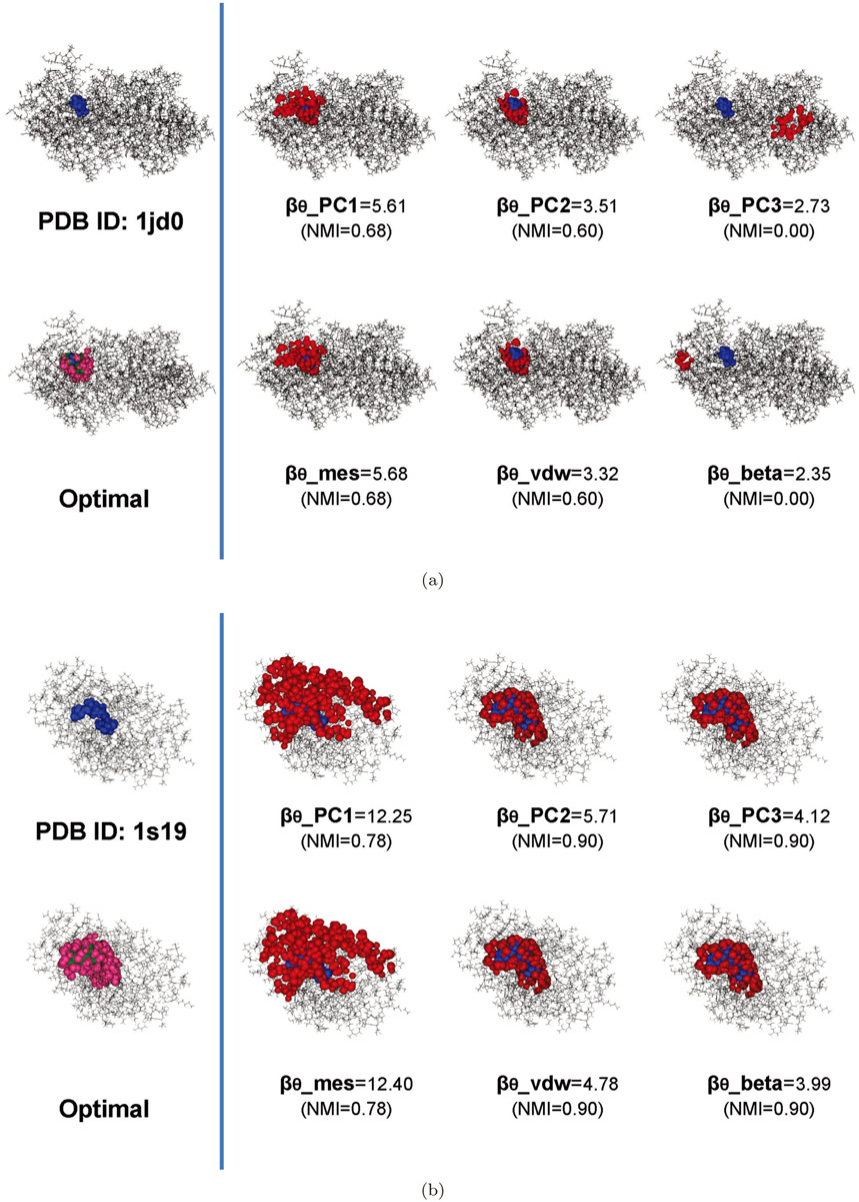
The optimal and recognized pockets of the PDB models. (a) PDB ID: 1jd0 (carbonic anhydrase XII—acetazolamide(18 atoms) complex) (b) PDB ID: 1s19 (vitamin D nuclear receptor-calcipotriol(70atoms) complex). The atoms are the colored receptor in black, the ligand in blue, the optimal pocket in pink, and the recognized pocket in red.

Fig. 14 shows some examples of recognized pockets using the six L-descriptor types from the two receptors (PDB accession codes: 1jd0 and 1s19) in the Astex Diverse Set. The *NMI* metric of each recognized pocket is shown in the figure. Fig. 14(a) shows 1jd0 (the carbonic anhydrase XII-acetazolamide complex), which has a small ligand consisting of 18 atoms. In this case, *β*_*θ*__*PC*3 and *β*_*θ*__*beta* are totally incorrect in that any atom of the optimal pocket is not contained within the recognized pocket. The value of *β*_*θ*__*PC*1 and *β*_*θ*__*mes* computes relatively large pockets compared to the size of the optimal pocket. Fig. 14(b) shows 1s19 (the vitamin D nuclear receptor-calcipotriol complex), which has a large ligand consisting of 70 atoms. In this case, *β*_*θ*__*PC*1 and *β*_*θ*__*mes* computes pockets that are too large compared to the size of the optimal pocket. In both cases, the *β*_*θ*__*vdw* and the *β*_*θ*__*PC*2 consistently predict good quality pockets.

**Fig 15:**
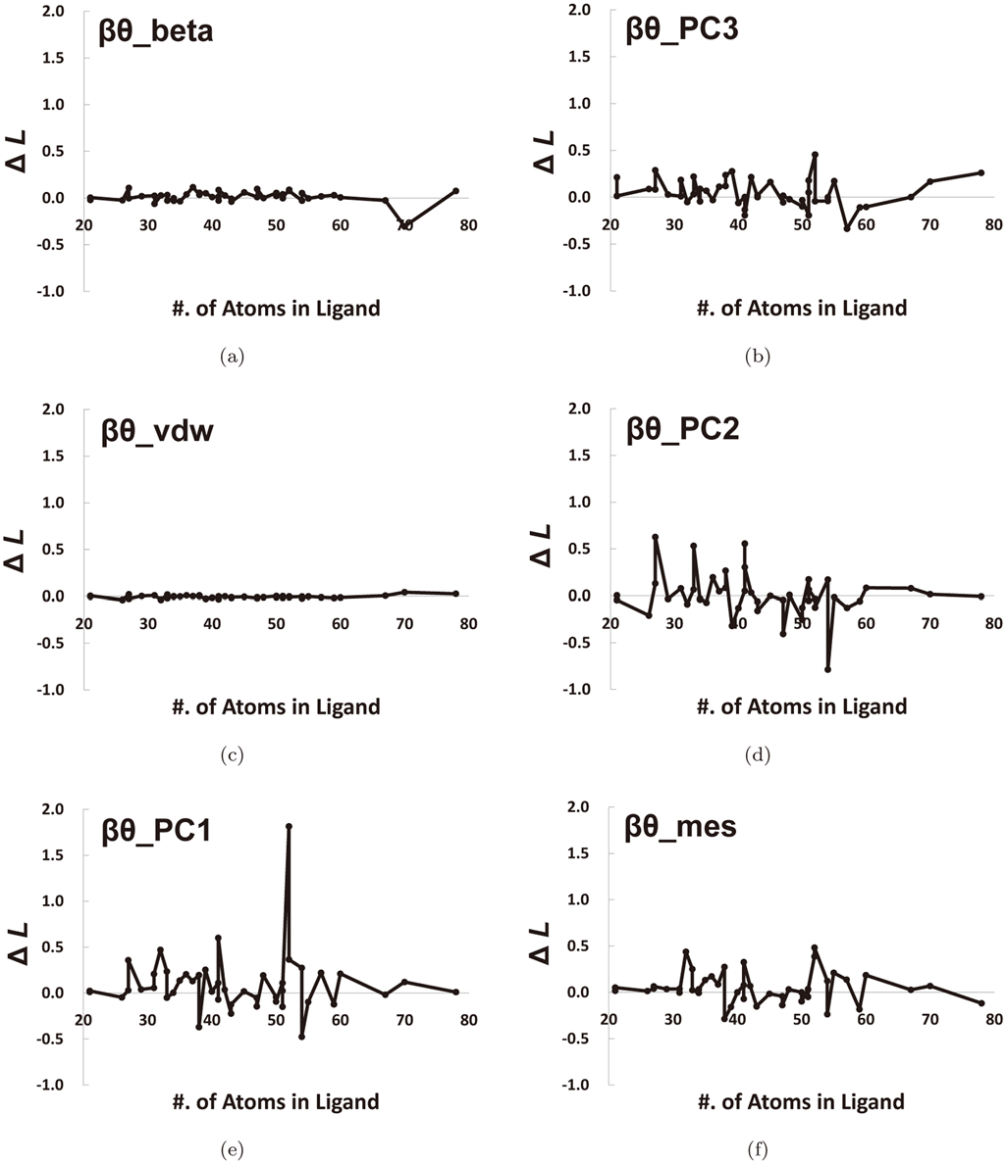
Difference in the *β*_*θ*_ values by change of the ligand conformation. $\Delta L=\beta_{\theta_{X}}^{bound}$—$\beta_{\theta_{X}}^{opt}$ (ie, Δ*L* = (*β*_*θ*_ of the bound ligand)−(*β*_*θ*_ of the ligand with minimum energy)).

Let *l* ^*bound*^ and *l* ^*opt*^ be the ligand conformations found in the crystal structure and in the minimum energy conformation, respectively. Let $\beta_{\theta_{X}}^{Y}$ be the value of *l* ^*Y*^ for the L-descriptor type *X* of *l* ^*opt*^, where *X* is one of the six L-descriptor types and *Y* ∈ {*bound*,*opt*}. Fig. 15 shows the graphs for $\Delta L=\beta_{\theta_{X}}^{bound}$—$\beta_{\theta_{X}}^{opt}$ for the Astex Diverse Set. Note that the graph of *β*_*θ*__*vdW* and *β*_*θ*__*beta* show less fluctuations compared to the other four; this implies that they are less sensitive to ligand conformation and less affected by the flexibility of the ligand. The fluctuation in the four graphs other than Fig. 15(a) and (c) implies that the corresponding L-descriptors are very sensitive to the ligand’s flexibility. From the experiment, we conclude that *β*_*θ*__*vdW* is optimal in that it yields a consistently good performance regardless of ligand size and conformational change.

### Benchmark

We benchmarked the proposed method against the STP (surface triplet propensities) algorithm [67] for recognizing the pockets of each protein in the Astex Diverse Set after removing the drug-like compounds. The STP algorithm assigns a score, called a patch score ranging between 0 to 100, to each and every atom of a protein. A higher value of the score implies that the atom has a higher probability to belong to a pocket. The STP algorithm selects those atoms whose scores are greater than a given threshold as the constituent of a predicted pocket. Thus, a higher patch score as a threshold selects fewer atoms than a lower one does. Be aware that the proposed method of this paper produces multiple components of boundary mesh where each can be a pocket candidate.

**Fig 16:**
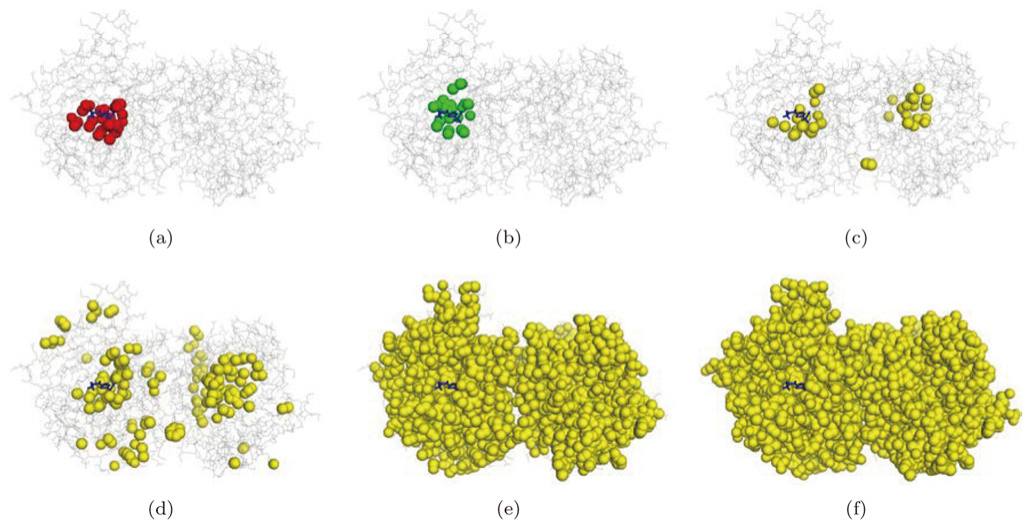
The visualization of pockets (PDB accession code: 1jd0). (a) The optimal pocket, (b) the best matched component produced by the proposed method, (c), (d), (e), and (f) are the atoms recognized by the STP method for the threshold values 80, 60, 40, and 20, respectively.

**Fig 17:**
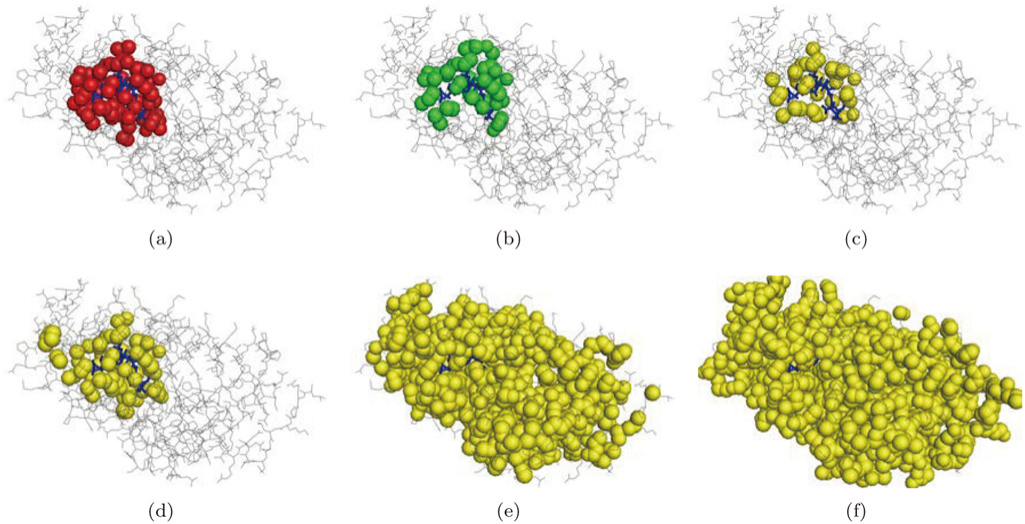
The visualization of pocket (PDB accession code: 1s19). (a) The optimal pocket, (b) the best matched component produced by the proposed method, (c), (d), (e), and (f) are the atoms recognized by the STP method for the threshold values 80, 60, 40, and 20, respectively.

Fig. 16 shows the optimal pocket (Fig. 16(a)), the pocket computed by the proposed method (Fig. 16(b)), and the one by the STP method (Fig. 16(c) through (f)) for a protein (PDB Accession code: 1jd0). The bound compound is visualized as a set of blue sticks (for the reference purpose), the atoms belonging to pockets are visualized as colored balls, and the rest of the protein structure is visualized as gray line segments. The red balls in Fig. 16 (a) are the atoms of the optimal pocket; The green balls in Fig. 16 (b) are the atoms of the best matched component produced from the proposed algorithm; The yellow balls in Fig. 16 (c), (d), (e), and (f) are the atoms recognized by the STP method for the threshold values 80, 60, 40, and 20, respectively. Fig. 17 shows another example (PDB Accession code: 1s19). Experiments with other proteins show similar results.

The examples above show that the proposed method seems very powerful without any parameters and perhaps better than the STP method. This claim is asserted by the following benchmark consisting of two types of tests. The first test type is the following. The proposed method selects the best five pocket candidates and the STP method selects atoms based on a threshold. We also select atoms at random for the reference where each random atom set has the size identical to the set produced by the STP method for each threshold value. Then, all atoms of each method forms one set, without processing to identify components where a “component” is a cluster of molecular boundary atoms which are topologically connected to each other. In this regard, we refer to this test type as “Without (component).”

The second test type is identical to the first except that the atoms in the atom set of each method are clustered together by the connectivity between the atoms. Then, the best matched component is used for the test. In this regard, we refer to this test type as “With (component).”

The following notations are for the “Without” case:

- *A* ^*Beta*^: The set of atoms in the five largest candidate sets by the proposed method.
- *A* ^*STP*^: The set of atoms by the STP method corresponding to each threshold *τ* whose value is determined from 0 to 95 by the increment of 5.
- *A* ^*Random*^: The set of randomly selected atoms where the *n*(*A* ^*Random*^) = *n*(*A* ^*STP*^) where *n*(*A*) is the number of elements of *A*.

The following notations are for the “With” case:

- *A* ^Beta*^: The best matched atom set to the optimal pocket by the proposed method.
- *A* ^STP*^: The best matched component (of atom set) defined by clustering the atoms in *A* ^*STP*^.
- *A* ^Random*^: The best matched component of *A* ^*Random*^.

**Fig 18:**
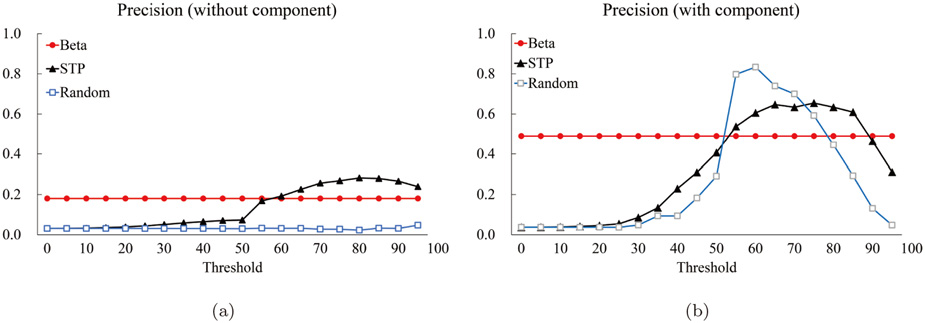
The precision graphs. **The red circle corresponds to the proposed method. The black triangle and blue square correspond to the average value (of the 85 structures of the Astex Diverse Set) for the STP and Random methods for each threshold value, respectively. The horizontal and the vertical axes denote the thresholds and the computed values of precision, respectively.** (a) Precision for “Without (component)” and (b) one for “With (component).”

**Fig 19:**
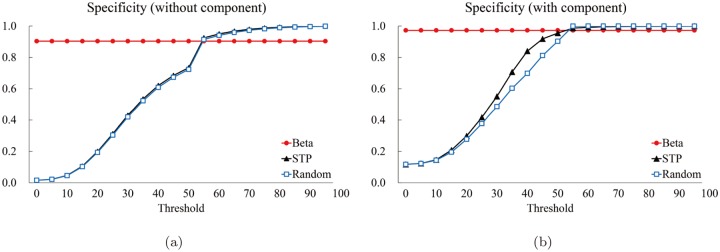
The specificity graphs. **The red circle corresponds to the proposed method. The black triangle and blue square correspond to the average value (of the 85 structures of the Astex Diverse Set) for the STP and Random methods for each threshold value, respectively. The horizontal and the vertical axes denote the thresholds and the computed values of specificity, respectively.** (a) Specificity for “Without (component)” and (b) one for “With (component).”

**Fig 20:**
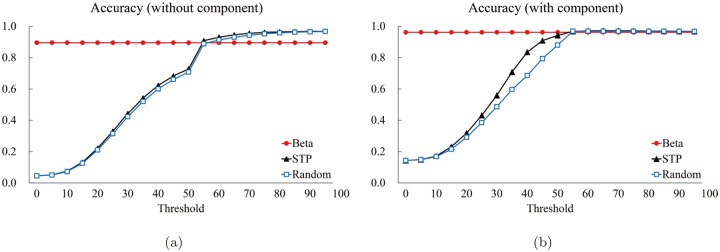
The accuracy graphs. **The red circle corresponds to the proposed method. The black triangle and blue square correspond to the average value (of the 85 structures of the Astex Diverse Set) for the STP and Random methods for each threshold value, respectively. The horizontal and the vertical axes denote the thresholds and the computed values of accuracy, respectively.** (a) Accuracy for “Without (component)” and (b) one for “With (component).”

**Fig 21:**
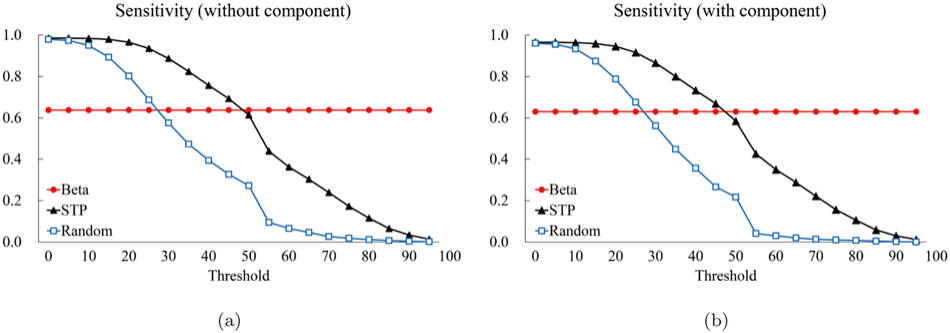
The sensitivity graphs. **The red circle corresponds to the proposed method. The black triangle and blue square correspond to the average value (of the 85 structures of the Astex Diverse Set) for the STP and Random methods for each threshold value, respectively. The horizontal and the vertical axes denote the thresholds and the computed values of sensitivity, respectively.** (a) Sensitivity for “Without (component)” and (b) one for “With (component).”

**Fig 22:**
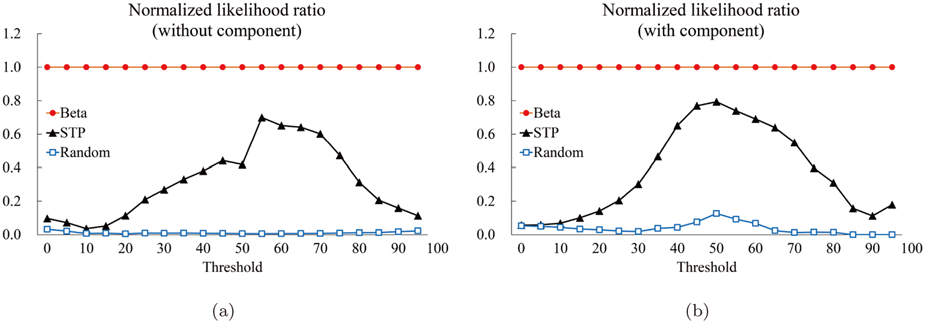
The normalized likelihood ratio graphs. **The red circle corresponds to the proposed method. The black triangle and blue square correspond to the average value (of the 85 structures of the Astex Diverse Set) for the STP and Random methods for each threshold value, respectively. The horizontal and the vertical axes denote the thresholds and the computed values of likelihood ratio, respectively.** (a) The normalized likelihood ratio for “Without (component)” and (b) one for “With (component).”

We computed the five measures: The precision *P* (Fig. 18), the specificity *SP* (Fig. 19), the accuracy *AC* (Fig. 20), the sensitivity *S* (Fig. 21), and the normalized likelihood ratio *LR* (Fig. 22).

Fig. 18(a) shows the graphs of the precision for the three methods for “Without.” The horizontal axis denotes the threshold and the vertical axis the computed precision value. Note the the proposed method, shown by the red solid circle labeled by “Beta,” is constant, independent of the threshold. On the other hand, the STP (the black triangle) and the Random (the blue rectangle) methods heavily depends on the threshold value. It seems that the STP method behaves better than the proposed method if the threshold is sufficiently big, say ≥ 60. No surprise to see the Random method behaves the worst.

Fig. 18(b) shows the precision graph for “With” component case. It is interesting to see that both STP and Random behave very well from the precision point of view if the threshold is big enough. Surprisingly the Random method shows the best precision for the range approximately between 55 and 70: It seems that this is because the Random method forms several component where each consists of relatively few atoms than the other two methods and some of the member atoms belong to the true pocket.

Fig. 19(a) shows the graphs for the specificity for the “Without” case. It is interesting that the STP and Random methods are surprisingly close and both produces slightly higher values than the proposed method where the threshold is bigger than (approximately) 60. The “With” case, Fig. 19(b), shows a similar behavior but all three methods are similar for bigger threshold values. Fig. 20 are the accuracy graphs which show patterns very similar to the specificity graphs. The similarity between the specificity and the accuracy is because there are significantly more atoms not belonging to the true pocket than the number of atoms belonging to the true pocket.

Fig. 21 shows the sensitivity graphs. While the proposed method (the red circle) shows a constant behavior, the STP method shows a decreasing pattern as the threshold increases and the two curves crosses approximately at the threshold of 50. It is obvious that the STP curve is monotonic because *A* ^*STP*^(*τ* = *τ*_1_) ⊆ *A* ^*STP*^(*τ* = *τ*_2_), *τ*1 > *τ*_2_. As is expected, the graph of Random method is lower than the STP method. It is important to note that both Fig. 21(a) and (b) are very close to each other. This is because, regardless which method is used, the best matching component contains most of the atoms of the optimal pocket.

Fig. 22 shows the normalized likelihood graphs. Note that the proposed method outperforms the others independent of the threshold value.

**Fig 23:**
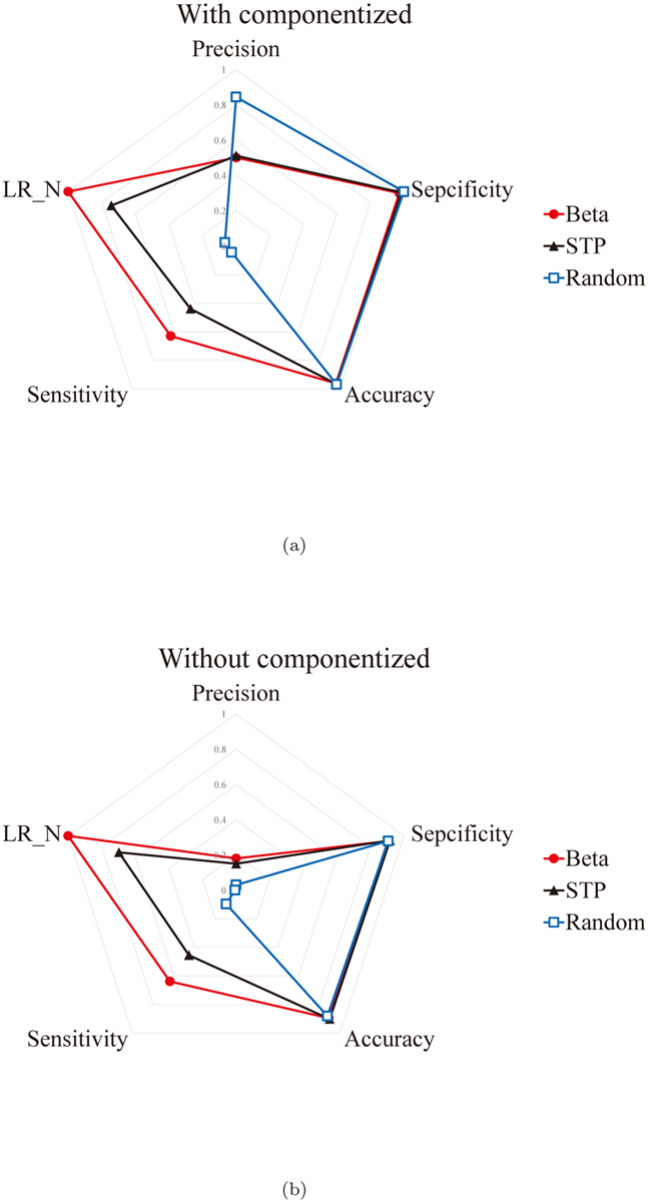
The radar charts of the proposed algorithm, the STP algorithm, and the Random method for the five statistical measures. (a) The case corresponding to the five best pockets recognized by the proposed algorithm, and (b) the case corresponding to the best pocket recognized by the proposed algorithm.

We performed another test as follows. Let *A* ^*Beta*^ be the set of all atoms belonging to the best five pockets recognized by the proposed algorithm. Let *A* ^*STP*^′^^ be the set of *n*(*A* ^*Beta*^) atoms recognized by the STP method. This means that we collect the best *n*(*A* ^*Beta*^) atoms from the one with the highest patch score to the ones with lower score, without considering the threshold. Let *A* ^*Random*^′^^ be the set of *n*(*A* ^*Beta*^) atoms randomly selected. Fig. 23(a) shows the distribution of the five statistical measures for the three methods. Suppose that we find the best matching component among the five pockets recognized by the proposed algorithm and let *A* ^Beta*^ be the set of the atoms belonging to this pocket. Let *A* ^*STP*^′^*^ and *A* ^*Random*^′^*^ be the sets of *n*(*A* ^Beta*^) atoms recognized by the STP and the Random methods, respectively. Fig. 23(b) shows the distribution of the five statistical measures for the three methods with the three atom sets *A* ^Beta*^, *A* ^*STP*^′^*^ and *A* ^*Random*^′^*^.

From the analysis above, we claim that the proposed method is better than the STP method in that it produces better quality pocket and is more robust.

## Conclusion

This paper proposes a parameter optimization for a pocket recognition algorithm based on the recent theory of the beta-shape, which is a derivative structure of the Voronoi diagram of atoms in a molecule. The parameter optimization was done by considering the ligand shape, thus called the L-descriptor, in the pocket recognition process so that the recognized pocket is ligand-specific.

We examined six types of L-descriptor for ligands: the minimum enclosing sphere, the three principal axes of the principal component analysis, the van der Waals volume, and the beta-shape volume. From the experiment using the Astex Diverse Set containing 85 complexes of proteins with ligands and various statistical measures based on the confusion matrix, the L-descriptor based on the van der Waals volume showed the best and consistent performance throughout the entire range of the ligand size. The van der Waals volume also showed a consistent result over different ligand conformations. In conclusion, we claim that the van der Waals volume is the optimal shape descriptor of ligands for pocket recognition algorithms based on the beta-shape using a spherical probe representing the ligands. The claim is verified by a benchmark test against the STP algorithm using the Astex Diverse Set. The code for the proposed pocket algorithm will be included in the powerful BetaVoid program for extracting void features of molecules [68].

## Supporting Information

S1 Table The definition of symbols. (PDF)
