# Supplementary material for: Optimal Ligand Descriptor for Pocket Recognition Based on the Beta-Shape
Source: PLoS One. 2015 Apr 2;10(4):e0122787. doi: 10.1371/journal.pone.0122787 (PMC4383629; doi:10.1371/journal.pone.0122787)
Supplement: S1 Table — (PDF) [file pone.0122787.s001.pdf]

# Supporting Information Table S1

Table S1: The summary of symbol definitions.

| | | | |
|---|---|---|---|
| $\beta$ | Probe radius | $d(q, M^R)$ | The minimum distance between $q$ and $\partial M^R$ |
| $\pi$ | A pherical probe | $d(q, M^L)$ | The minimum distance between $q$ and $\partial M^L$ |
| $\beta_\theta\_mes$ | The radius of the enclosing sphere | $IIF^\infty$ | The mid-surface between receptor and ligand |
| $\beta_\theta\_PC1$ | The Euclidean norm of the longest vector of principle component analysis (PCA) | $IIF$ | The trimmed surface of $IIF^\infty$ |
| $\beta_\theta\_PC2$ | The Euclidean norm of the 2nd longest vector of PCA | $B$ | The atoms on the receptor boundary |
| $\beta_\theta\_PC3$ | The Euclidean norm of the shortest vector of PCA | $\Pi$ | The optimal pocket |
| $\beta_\theta\_vdW$ | The radius of the sphere whose volume is $Vol(vdW)$ | $\Pi^c$ | $B - \Pi$ |
| $\beta_\theta\_beta$ | The radius of the sphere whose volume is $Vol(beta)$ | $\widehat{\Pi}$ | The recognized pocket |
| $Vol(vdW)$ | The volume of the vdW-model | $\widehat{\Pi^c}$ | $B - \widehat{\Pi}$ |
| $M^R$ | A receptor | $NMI$ | Measure of information transmission |
| $M^L$ | A ligand | $LR$ | Likelihood ratio |
| $\partial$ | Boundary operator | | |
